# Comparative Genomic Analysis of Two *Vibrio toranzoniae* Strains with Different Virulence Capacity Reveals Clues on Its Pathogenicity for Fish

**DOI:** 10.3389/fmicb.2017.00086

**Published:** 2017-01-30

**Authors:** Aide Lasa, Cynthia J. Gibas, Jesús L. Romalde

**Affiliations:** ^1^Departamento de Microbiología y Parasitología, CIBUS-Facultad de Biología, Universidade de Santiago de CompostelaSantiago de Compostela, Spain; ^2^Department of Bioinformatics and Genomics, The University of North Carolina at CharlotteCharlotte, NC, USA

**Keywords:** *Vibrio toranzoniae*, fish pathogen, genomic comparison, pathogenicity islands, virulence factors

## Abstract

*Vibrio toranzoniae* is a Gram-negative bacterium of the Splendidus clade within the *Vibrio* genus. *V. toranzoniae* was first isolated from healthy clams in Galicia (Spain) but recently was also identified associated to disease outbreaks of red conger eel in Chile. Experimental challenges showed that the Chilean isolates were able to produce fish mortalities but not the strains isolated from clams. The aim of the present study was to determine the differences at the genomic level between the type strain of the species (CECT 7225^T^) and the strain R17, isolated from red conger eel in Chile, which could explain their different virulent capacity. The genome-based comparison showed high homology between both strains but differences were observed in certain gene clusters that include some virulence factors. Among these, we found that iron acquisition systems and capsule synthesis genes were the main differential features between both genomes that could explain the differences in the pathogenicity of the strains. Besides, the studied genomes presented genomic islands and toxins, and the R17 strain presented CRISPR sequences that are absent on the type strain. Taken together, this analysis provided important insights into virulence factors of *V. toranzoniae* that will lead to a better understanding of the pathogenic process.

## Introduction

Members of *Vibrionaceae* family are a predominant group of Gram-negative bacteria found in aquatic environment (Thompson et al., 2004). This bacterial group live as planktonic forms in the water column or associated in biofilms or with host organisms as pathogenic, commensal or mutualistic bacteria. *Vibrio* is the largest genera within this family with at least 130 recognized species (http://www.bacterio.net/vibrio.html). Many *Vibrio* species are recognized as pathogens of different organisms, including humans and aquatic animals. For instance, several species of the Splendidus clade, that comprises 17 species (Romalde et al., 2014; Pérez-Cataluña et al., 2016), are causing significant losses in the aquaculture industry worldwide.

*Vibrio splendidus* has been described as affecting fish species such as the turbot *Scophtalmus maximus* (Gatesoupe et al., 1999) but also, associated with mortalities of different molluscan species as scallops, *Pecten maximus* (Nicolas et al., 1996), *Pactinopecten yessonensis* (Liu et al., 2013) and *Argopecten purpurartus* (Rojas et al., 2015), oysters, *Crassostrea gigas* (Le Roux et al., 2002; Domeneghetti et al., 2014) and *Crassostrea virginica* (Elston and Leibovitz, 1980), and mussels (*Mytilus galloprovincialis*; Domeneghetti et al., 2014). Even though the virulence mechanisms involved in the pathogenesis of the *V*. *splendidus*-related species are not well-characterized, some virulence factors have been identified such as the invasion porine OmpU (Duperthuy et al., 2010), the metalloprotease Vsm (Le Roux et al., 2007; Binesse et al., 2008) and the invasive vesicular serine protease Vsp (Vanhove et al., 2014).

The virulence of pathogenic *Vibrio* strains isolated from molluscs has been shown to be related to their ability to produce extracellular products (Elston and Leibovitz, 1980; Labreuche et al., 2006; Hasegawa et al., 2008). For instance, *Vibrio lentus* has been recognized as a potential pathogen that caused skins lesions, colonize the internal organs and induce mortality in octopus (Farto et al., 2003). On subsequent studies (Farto et al., 2006), the presence of a lethal extracellular protease was detected in the ECP (extracellular products) of *V. splendidus*-*V. lentus* related group.

*V. kanalaoe* is also considered as a potential pathogen for aquatic animals including fish and crustaceans (Austin et al., 2005). Experimental infections of this species in *C. gigas* produced extensive lesions in the adductor muscle (Gay et al., 2004). Unfortunately, no clear discrimination between *V. kanaloae* and *V. pomeroyi* was possible, even using a polyphasic approach to identify the strains. Another member of the Splendidus clade, *Vibrio celticus*, showed potential pathogenic activity for adult clams (*R. phillipinarum* and *R. decussatus*) in virulence assays (Beaz-Hidalgo et al., 2010).

*V. toranzoniae* was first isolated from healthy clams (*R. phillipinarum* and *R. decussatus*) in Galicia (Spain) and designated as a new member of the Splendidus clade (Lasa et al., 2013). Further studies (Gulla et al., 2015; Kwan and Bolch, 2015) indicated that the geographical and host distribution of this species could be wider than expected. In fact, a group of strains, identified as *V. toranzoniae* using a polyphasic approach, were isolated from cultured red conger eel (*Genypterus chilensis*) in Chile during an episode of mortalities at one aquaculture rearing system (Lasa et al., 2015). Inoculation experiments in turbot of the Chilean isolates and the type strain of the species (CECT 7225^T^) demonstrated the pathogenic potential for fish of the strains isolated from red conger eel but not for the original strains isolated from clams.

The aim of the present study was to determine the differences, at the genome level, between the clam and Chilean isolates. In order to elucidate the genomic base of the pathogenic potential of *V*. *toranzoniae* species, the type strain CECT 7225^T^ and a representative strain of the Chilean isolates, R17, were selected for the genome comparison and the identification of virulence factors. The identification of putative pathogenicity islands common in the *Vibrio* genus was also evaluated.

## Materials and methods

### Genomic DNA extraction and sequencing

DNA extraction and sequencing project of strain R17 was performed following the protocol described previously for the type strain of the species (Lasa et al., 2016). Briefly, CECT 7225^T^ and R17 strains were routinely cultured in marine agar (MA; Difco) 24 h at 25°C. Genomic DNA was extracted using the QIAamp DNA minikit (Qiagen), according to the manufacturer's protocol. The purified DNA was used to prepare a library, following the Illumina TruSeq DNA sample prep protocol (Illumina, San Diego, CA). The genomes of *V*. *toranzoniae* strains were sequenced at David H. Murdock Research Institute (DHMRI) of the University of North Carolina (Kannapolis, North Carolina), using a HiSeq 2500 sequencing technology (Illumina) with 2 × 100-bp paired-end reads.

### Genome assembly, annotation, and analysis of the *Vibrio toranzoniae* genomes

The Illumina reads were analyzed for quality control using FASTQC (Brabaham Bioinformatics). Reads were trimmed and filtered to remove adapters and low quality bases, using Trimmomatic 0.32 (Bolger et al., 2014) program. The remaining reads were used for the genome assembly, performed with the SPAdes 3.6.1 the novo assembler tool (Nurk et al., 2013), and QUAST (Gurevich et al., 2013) software was used to evaluate the assembly.

The draft genome of the type strain was annotated using the NCBI Prokaryotic Genome Annotation Pipeline (PGAP; Angiuoli et al., 2008). Additionally, the genomes were analyzed on the Rapid Annotations using Subsystems Technology (RAST) server (Overbeek et al., 2014). CRISPRfinder tool (Grissa et al., 2007) was used to assess the presence of CRISPR repeats in both genomes.

A sequenced-based comparison analysis was performed using the RAST annotation server and Blast Ring Image Generator (BRIG; Alikhan et al., 2011) was used to obtain a genomic map showing similarity percentages. The upper and lower identity thresholds were set at 90 and 70%, respectively.

The presence of recognized virulence factors in other fish pathogens was determined. Identification of Genomic Islands (GIs) in the genomes of *V. toranzoniae* strains was performed by comparative analysis against some of the main fish pathogenic *Vibrio* species including *V. splendidus, V. vulnificus*, and *V. anguillarum* with IslandViewer (Waack et al., 2006; Langille et al., 2008; Dhillon et al., 2015) which uses the IslandPick, SIGI-HMM and IslandPath-DIMOB tools for the prediction.

### Genome deposit in public databases

The draft genome sequencing project of *Vibrio toranzoniae* strain CECT 7225^T^ numbers LMXU00000000. Additionally, annotated contigs of the type strain are available under the accession numbers LMXU01000001-LMXU01000165 (Lasa et al., 2016). The Whole Genome Shotgun project of R17 has been deposited at DDBJ/ENA/GenBank under the accession MRTB00000000. The version described in this paper is version MRTB01000000.

## Results

### Genomic features

The sequencing experiments of CECT 7225^T^ and R17 strains yielded 23,169,360 and 23,940,108 reads and the final draft assemblies contained 165 and 156 contigs, respectively, for each strain. The first draft genome summarizes 4,537,316 bp and the second 4,590,695 bp (Table 1).

**Table 1:** **Genome statistics for ***V. toranzoniae*** CECT 7225^**T**^ and R17**.

**Attribute**	**CECT 7225^T^^a^**	**R17**
Reads	23,169,360	23,940,108
N50	221,494	242,107
Genome size (bp)	4,537,316	4,590,695
G+C Content	43.9%	43.8%
Coding sequences	4068	4075
RNA genes	140	144
CRISPR repeats	–	2

a *data from Lasa et al. (2016)*.

The G+C content of *V*. *toranzoniae* CECT 7225^T^ was 43.9 mol%. A total of 4058 protein-coding sequences were predicted using RAST annotation server and 140 were RNA genes. Eighty one percent of coding sequences were assigned to putative functions, while 19% remained as hypothetical proteins. On the other hand, the G+C content of *V*. *toranzoniae* R17 strain was 43.8 mol%. The annotation performed on the RAST annotation server revealed 4075 protein-coding sequences and 144 RNA genes. Of the total, 80.8% of the coding sequences were assigned to putative functions and 19.2% remained as hypothetical proteins. The search of CRISPR repeats showed the presence of two sequences in the R17 strain, while the type strain lacked these sequences (Table 1).

**Figure 1:**
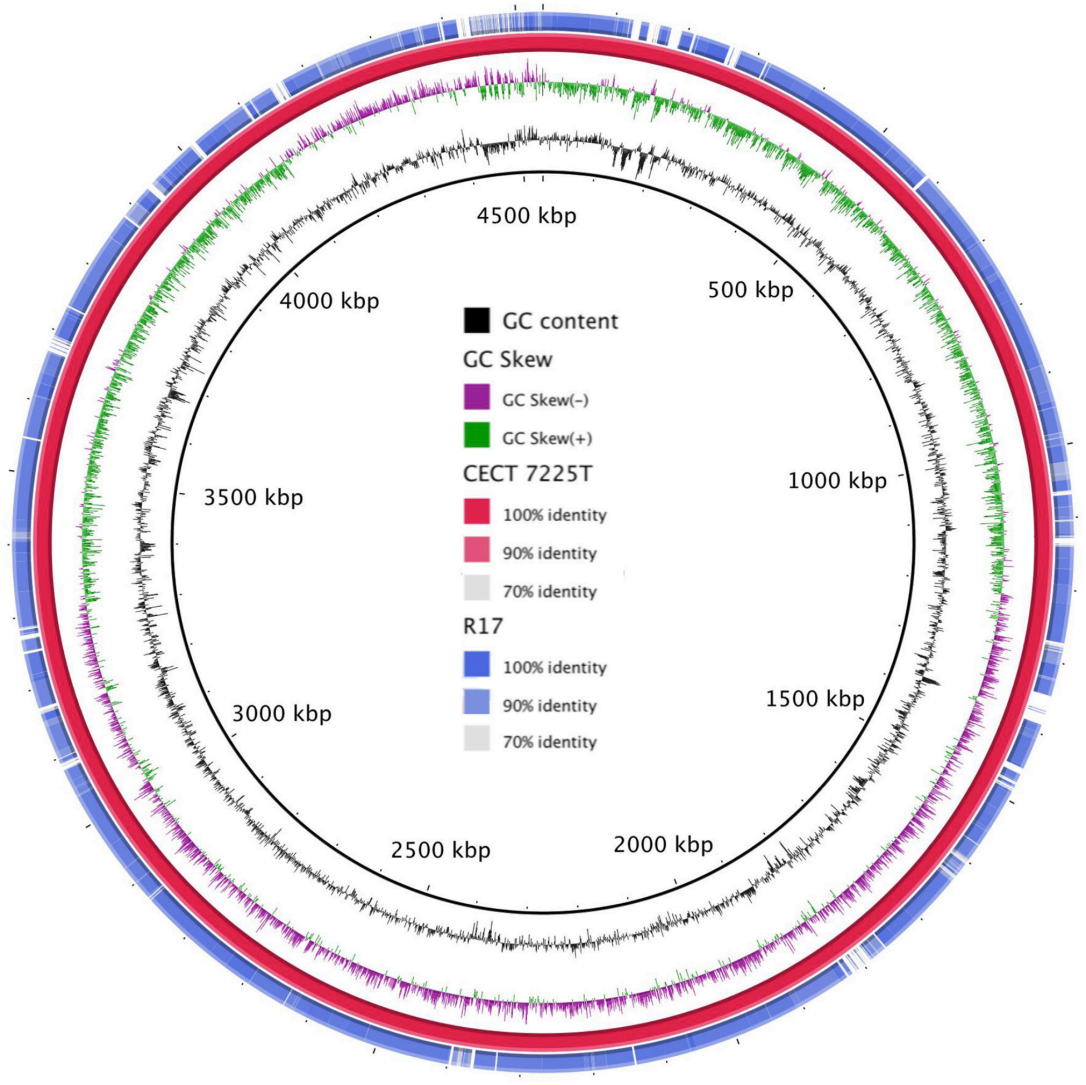
**Graphical map of the BLASTN comparison of the two ***V. toranzoniae*** draft genomes**. From center to outside: GC Content (ring 1), GC skew (ring 2), *V. toranzoniae* CECT 7225^T^ (ring 3), and *V. toranzoniae* R17 (ring 4).

The genomic map revealed a high homology between the type strain and R17 strain (Figure 1). Regions of major differences between both strains contained mobile elements, phage genes and virulence factors.

### Genomic and pathogenicity islands of the *V. toranzoniae* genomes

In the *Vibrio* species analyzed, GIs could be identified by the SIGI-HMM and IslandPath-DIMOB methods, but not using the IslandPick method. In both genomes, the highest number of GIs were found using SIGI-HMM software. The highest number of proteins within identified GIs were found when comparing against *V. anguillarum* (443) for the two studied genomes (Table 2). Interestingly, among the identified proteins different virulence factors were found, namely hemolysins, toxins, or iron acquisition system.

**Table 2:** **Number of identified GIs and proteins in both genomes**.

**Comparison with:**	**CECT 7225**^**T**^			**R17**		
	**SIGI-HMM**	**IslandPath-DIMOB**	**Identified proteins**	**SIGI-HMM**	**IslandPath-DIMOB**	**Identified proteins**
*V. splendidus*	30	10	416	26	6	265
*V. vulnificus*	28	8	420	24	4	263
*V. anguillarum*	27	11	443	23	5	283

### Pathogenic and virulence related features

Based on established studies, we scanned both *V. toranzoniae* genomes for virulence-related features that could explain the different ability of these strains to produce disease in fish (summarized in Table 3).

**Table 3:** **Summary of the virulence factors for both ***V. torazoniae*** strains**.

	**Virulence factors**	
	**CECT 7225^T^**	**R17**
**CELL WALL AND CAPSULE**		
dTDP-rhamnose synthesis	–	dTDP-4-dehydrorhamnose reductase
	dTDP-glucose 4,6-dehydratase	dTDP-glucose 4,6-dehydratase
	dTDP-4-dehydrorhamnose 3,5-epimerase	dTDP-4-dehydrorhamnose 3,5-epimerase
	Glucose-1-phosphate thymidylyltransferase	Glucose-1-phosphate thymidylyltransferase
Capsular polysaccharides biosynthesis and assembly	Polysaccharide export lipoprotein Wza	Polysaccharide export lipoprotein Wza
	Tyrosine-protein kinase Wzc	Tyrosine-protein kinase Wzc
	Oxidoreductase, short-chain dehydrogenase/reductase family	Oxidoreductase, short-chain dehydrogenase/reductase family
	–	Oligosaccharide repeat unit polymerase Wzy
	Low molecular weight protein-tyrosine-phosphatase Wzb	Low molecular weight protein-tyrosine-phosphatase Wzb
**IRON ACQUISITION**		
Siderophore assembly kit	–	Siderophore synthetase large component, acetyltransferase
	–	Siderophore synthetase component, ligase
	–	ABC-type hemin transport system, ATPase component
	–	Periplasmic hemin-binding protein
	–	Siderophore synthetase small component, acetyltransferase
	–	Hemin ABC transporter, permease protein
	–	Siderophore biosynthesis protein, monooxygenase
	–	Putative ABC iron siderophore transporter, fused permease and ATPase domains
	–	TonB-dependent hemin, ferrichrome receptor
Siderophore aerobactin	Aerobactin siderophore receptor IutA	Aerobactin siderophore receptor IutA
	–	L-lysine 6-monooxygenase [NADPH], aerobactin biosynthesis protein IucD
	–	Citrate 6-N-acetyl-6-N-hydroxy-L-lysine ligase alpha subunit, aerobactin biosynthesis protein IucA
	–	N6-hydroxylysine O-acetyltransferase, aerobactin biosynthesis protein IucB
	–	Citrate:6-N-acetyl-6-N-hydroxy-L-lysine ligase alpha subunit, aerobactin biosynthesis protein IucA
	Ferric aerobactin ABC transporter, permease component	Ferric aerobactin ABC transporter, permease component
	Ferric aerobactin ABC transporter, periplasmic substrate binding protein	Ferric aerobactin ABC transporter, periplasmic substrate binding protein
	Ferric aerobactin ABC transporter, ATPase component	Ferric aerobactin ABC transporter, ATPase component
**TOXINS**		
	Haemolysin	Haemolysin
	RTX toxin (probable)	RTX toxin (putative)

Related to these virulence factors, we found differences in the gene content of the systems involved in the synthesis of certain capsular polysaccharides. Thus, in the strain CECT 7225^T^ the gene of the Wzy polymerase is absent and also a reductase gene of the dTDP-rhamnose pathway.

**Figure 2:**
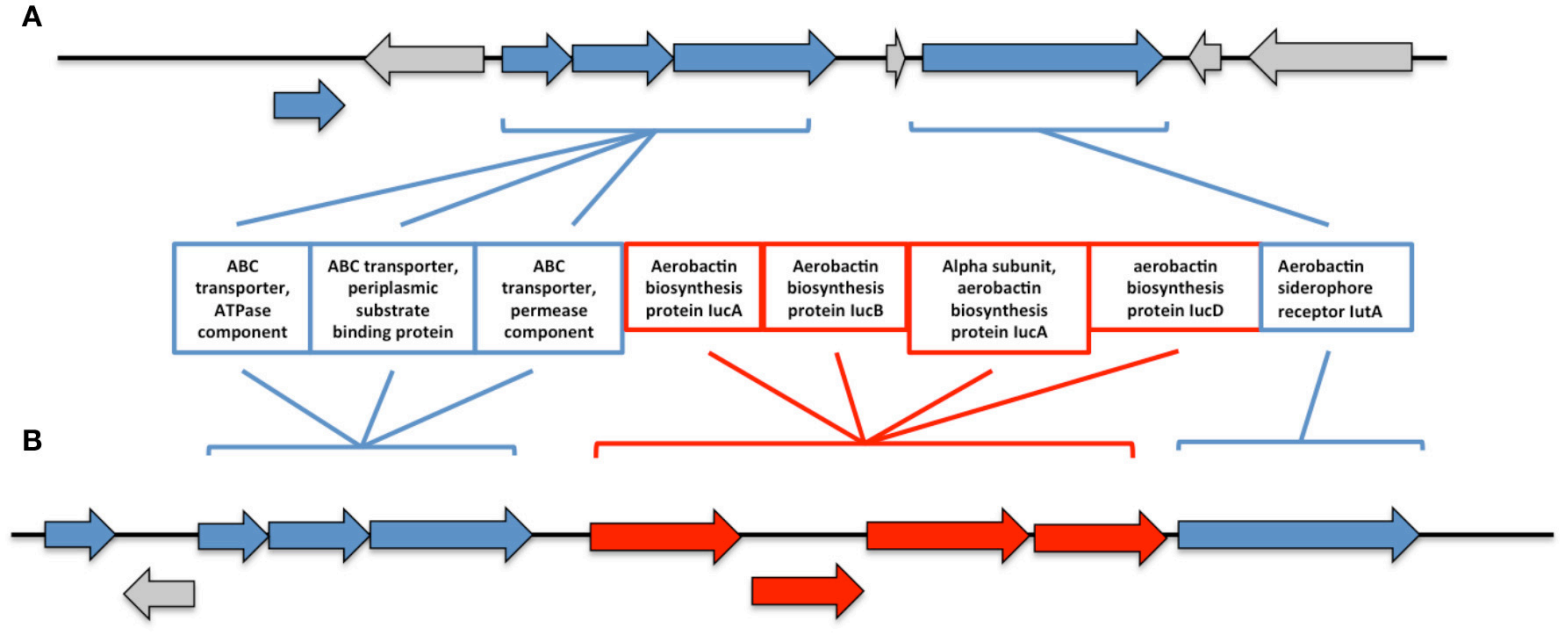
**Genomic region of the aerobactin system in the genomes of CECT 7225^**T**^ (A)** and R17 **(B)** strains. In red are indicated the genes that are absent on the type strain.

Additionally, we found significant differences in the iron metabolism between both genomes. In this sense, R17 strain posseses an entire cluster of genes for siderophore assembly kit that encodes ligase, acetyltransferases, monooxygenase, an ABC-type transport system and a ferrichrome receptor, which is absent in the CECT 7225^T^ strain. Besides, we found the presence of other iron uptake proteins in both genomes coding an aerobactin system, however in the CECT 7225^T^ genome the genes that encode the aerobactin are absent and only the transporter and receptor genes are present. The region of the aerobactin iron transport system observed in both genomes showed the absence of the genes IucABCD genes in the type strain (Figure 2). The predicted IucABCD proteins showed 70–82% identity to the corresponding proteins of other *Vibrio* species.

The presence of proteins with cytotoxic activity was assessed and in the genome of R17 strain a toxin was found and annotated as putative RTX toxin. Also in the CECT 7225^T^ genome this toxin was present but the protein was annotated as a probable toxin. The difference between these two proteins seems to be related to the length of the gene, which is shorter in the CECT 7225^T^ genome. Another virulence factor with cytotoxic activity that we found and is present in both strains was a haemolysin with high homology to other haemolysins present in the Splendidus clade species.

## Discussion

Our analysis depicted the first step to understand the pathogenic potential of *V. toranzoniae* for fish. The results obtained may explain the specificity of the species to produce disease in fish but not in clams, since the main virulence factors found here are related to iron uptake. However, it cannot be discard the pathogenic potential for molluscs and further experimental assays will be necessary.

The capsule is a recognized virulence factor that contributes to virulence in other *Vibrio* pathogens (Williams et al., 2014; Oliver, 2015). The proposed function of this factor is to protect the organism from phagocytosis, thus initiating the infection. Capsulated strains grow on solid media forming opaque colonies, while non-capsulated strains develop translucent colonies. Therefore, we have observed that R17 strain form opaque colonies and the type strain CECT 7225^T^ develop into translucent colonies when grown on solid media. The formation of translucent colonies of the CECT 7225^T^ strain can be explained by the loss of important proteins that are involved in the synthesis of capsular polysaccharides. The Wzy polymerase is required for the extension of polysaccharides of the capsule and also of the O-antigen (Islam and Lam, 2014). Additionally, the rhamnose synthesis pathway, which seems to be interrupted on CECT 7225^T^ strain, is involved in the synthesis of these capsular polysaccharides. Other virulence factor that would be crucial for the initial infection and disease development are the CRISPR arrays. The presence of CRISPR sequences in the R17 strain would allow this strain not only to resist the infection of virus but also to avoid the host immune system (Louwen et al., 2014) promoting the infection and the dissemination within the fish.

Genomic islands are large genomic regions that contain multiple genes, probably with horizontal transfer origin. GIs are classified based on the different functions they encode, including metabolic islands, degradation islands, symbiosis islands, and pathogenicity islands (Murphy and Boyd, 2008). Pathogenicity islands are unstable regions that exhibit virulence-related genes that are involved in virulence, antibiotic resistance and other adaptations to the infective process (Langille et al., 2010). Most of the annotated proteins within the GIs of the *V. toranzoniae* genomes were identified as hypothetical proteins, transposases, or ribosomal proteins and are present in all *Vibrio* (Lux et al., 2014). Important virulence factors were found within these predicted GIs, including haemolysin, RTX toxin or iron uptake systems.

Iron uptake systems and siderophores production allows fish pathogens to avoid the natural defense mechanisms of the fishes using iron-binding proteins (such as trasnferrin, lactoferrin, or ferritin). Interestingly, the siderophore assembly kit was present only in the R17 strain and the presence of this system could provide important advantages in regards to infection, pathogenicity, and resistance. In addition, the CECT 7225^T^ strain may have lost these advantages by losing the group of genes that encode the aerobactin, despite this strain are able to produce the transporter system and the receptor.

Cytotoxic activity may be possible through the codification of toxins. Homologs to putative RTX toxins have been identified in *V. vulnificus* and may play a role in cell adhesion and/or pathogenesis, and in *V*. *anguillarum* are major component of virulences. These toxins exhibit a cytotoxic pore-forming activity and the secretion occurs via type I secretion system. We found that the coding gene for this toxin is present in both strains, but the open reading frame of the CECT 7225^T^ strain appear to be disrupted. Other virulence factor that contributes to the cytotoxic activity is the haemolysin and both strains have these coding genes. In fact, these strains produce β haemolysis when grew on blood agar plates.

Other potential virulence factors, such as Vsm metalloproteases, Vsp proteases, or extracellular proteases, were not found within the annotated proteins or the GIs. However, cannot be discard the presence of this virulence related proteins among the genes that were not identified and they remained as hypothetical proteins.

## Conclusions

The genome-based comparison presented here reveal common basic features between the two *V*. *toranzoniae* strains. However the distribution of virulence factors was different between the isolates, especially those systems related to iron acquisition systems and capsule production. In general, these results are in congruence with the previous observations on the pathogenic ability of the isolates, although further *in vivo* analysis, including different aquatic animal models and the construction of mutants for the different virulence-related genes, are required to confirm the role of this factors in the pathogenic process.

## Author contributions

The experiments, data analysis, and manuscript writing were performed by AL and JR, while CG provided vital guidance and technical support.

### Conflict of interest statement

The authors declare that the research was conducted in the absence of any commercial or financial relationships that could be construed as a potential conflict of interest.
